# Natural language processing of radiology reports for identification of skeletal site-specific fractures

**DOI:** 10.1186/s12911-019-0780-5

**Published:** 2019-04-04

**Authors:** Yanshan Wang, Saeed Mehrabi, Sunghwan Sohn, Elizabeth J. Atkinson, Shreyasee Amin, Hongfang Liu

**Affiliations:** 1 Division of Biomedical Statistics and Informatics, Department of Health Sciences Research, Mayo Clinic, 200 1st ST SW, Rochester, MN, USA; 2 Division of Rheumatology, Department of Medicine, Mayo Clinic, 200 1st ST SW, MN, Rochester, USA; 3 Division of Epidemiology, Department of Health Sciences Research, Mayo Clinic, 200 1st ST SW, MN, Rochester, USA

**Keywords:** Fracture identification, Natural language processing, Radiology reports, Electronic health records

## Abstract

**Background:**

Osteoporosis has become an important public health issue. Most of the population, particularly elderly people, are at some degree of risk of osteoporosis-related fractures. Accurate identification and surveillance of patient populations with fractures has a significant impact on reduction of cost of care by preventing future fractures and its corresponding complications.

**Methods:**

In this study, we developed a rule-based natural language processing (NLP) algorithm for identification of twenty skeletal site-specific fractures from radiology reports. The rule-based NLP algorithm was based on regular expressions developed using MedTagger, an NLP tool of the Apache Unstructured Information Management Architecture (UIMA) pipeline to facilitate information extraction from clinical narratives. Radiology notes were retrieved from the Mayo Clinic electronic health records data warehouse. We developed rules for identifying each fracture type according to physicians’ knowledge and experience, and refined these rules via verification with physicians. This study was approved by the institutional review board (IRB) for human subject research.

**Results:**

We validated the NLP algorithm using the radiology reports of a community-based cohort at Mayo Clinic with the gold standard constructed by medical experts. The micro-averaged results of sensitivity, specificity, positive predictive value (PPV), negative predictive value (NPV), and F1-score of the proposed NLP algorithm are 0.930, 1.0, 1.0, 0.941, 0.961, respectively. The F1-score is 1.0 for 8 fractures, and above 0.9 for a total of 17 out of 20 fractures (85%).

**Conclusions:**

The results verified the effectiveness of the proposed rule-based NLP algorithm in automatic identification of osteoporosis-related skeletal site-specific fractures from radiology reports. The NLP algorithm could be utilized to accurately identify the patients with fractures and those who are also at high risk of future fractures due to osteoporosis. Appropriate care interventions to those patients, not only the most at-risk patients but also those with emerging risk, would significantly reduce future fractures.

## Introduction

Osteoporosis is an important public health issue, owing to the fact that a substantial proportion of the aging population will experience fractures associated with low bone mass [1]. According to World Health Organization (WHO), an estimated 10 million Americans over 50 years old already have osteoporosis [2], while over 33 million more have “osteopenia”, which is a reduction in bone density that can precede osteoporosis. The total number with low bone mass could reach 61 million by 2020 [3]. Likewise, the estimated 2 million osteoporosis-related fractures in 2005 could exceed 3 million by 2025, with an associated increase in costs from $16.9 billion to $25.3 billion annually [4]. It also has been shown that most of the population, besides elderly people, are at some degree of risk of osteoporosis-related fractures [5]. Accurate identification of fractures will help identify the patients with high risk of future fractures. Applying appropriate interventions to those patients would significantly reduce future fracture, and reduce the cost of care [5].

Significant amounts of information for identification of fractures are only available in a narrative format. Manually extracting such information from clinical narratives is time consuming and expensive. Fortunately, prevalence of Electronic Health Records (EHRs) makes automated fracture identification more feasible than before. EHR has provided new means to extract information through analysis of clinical diagnostic narratives. Radiology reports are one particularly rich source of clinical diagnostic information. Researchers have utilized Natural Language Processing (NLP) techniques to extract information from these reports [6]. NLP algorithms have been developed for automatic information extraction for a variety of diseases [7, 8], including appendicitis [9], pneumonia [10], thromboembolic diseases [11], and various potentially malignant lesions [12]. Most of these applications exploit manually designed rules based on medical experts’ knowledge and experience, which has been called rule-based NLP algorithms.

A few rule-based NLP algorithms have been proposed for the identification of fractures from radiology reports in the literature. Yadav et al. [13] developed a hybrid system of NLP and machine learning for automated classification of orbital fracture from emergency department computed tomography (CT) reports. Wagholikar et al. [14] used NLP rules to classify limb abnormalities from radiology reports using a clinician informed gazetteer methodology. VanWormer et al. [15] developed a keyword search system to identify patients who were injured because of tree stand falls during hunting seasons. Do et al. [16] used NLP in an application that extracts both the presence of fractures and their anatomic location. Grundmeier et al. [17] implemented and validated NLP tools to identify long bone fractures for pediatric emergency medicine quality improvement. However, few of these studies have well-defined skeletal site-specific fractures, and report specific rules for each of skeletal site-specific fractures from radiology reports.

In this study, we developed a rule-based NLP algorithm for identification of twenty skeletal site-specific fractures from radiology reports. We applied and tested the algorithm on a cohort at Mayo Clinic within a well-defined community, Rochester Epidemiology Project (REP) [18–20], with the gold standard constructed by medical experts.

## Method

## Study setting

The study was conducted at Mayo Clinic, Rochester MN. A fracture cohort of 1349 Mayo Clinic patients who were 18 years of age or older and experienced fractures in 2009–2011 was utilized in our study [21, 22]. In addition, we selected a control cohort of 2000 Mayo Clinic patients who lived in Olmsted County any time from 2008–2012, were 18 years of age or older in 2008, and had no evidence of having a fracture through their entire known follow-up in 2008–2017. Nurses with multiple years of experience abstracting fractures reviewed each subject’s entire patient record and created the gold standard. This study was approved by the institutional review board (IRB) for human subject research.

We utilized twenty skeletal site-specific fractures that have been used by the Osteoporosis Research Program at Mayo Clinic for over 30 years [21, 22]. These skeletal sites included ankle, clavicle, distal forearm, face, feet and toes, hand and figures, patella, pelvis, proximal femur, proximal humerus, ribs, scapula, shaft and distal femur, shaft and distal humerus, shaft and proximal radius/ulna, skull, sternum, tibia and fibula, vertebral body, and other spine. Since a single subject may have experienced multiple fractures, our study included a total of 2356 fractures in 1349 subjects.

Radiology notes, including general radiography reports (such as X-ray reports), computed tomography reports, magnetic resonance imaging reports, nuclear medicine radiology reports, mammography reports, ultrasonography reports, neuroradiology reports, were retrieved from the Mayo Clinic EHR warehouse for all the subjects.

For each fracture type, we randomly utilized 70% of the subjects in the fracture cohort as training data to develop the rule-based NLP algorithm, and the remaining 30% of the subjects in the fracture cohort with the identical number of subjects randomly sampled from the control cohort as testing data to evaluate the algorithm. The exact number of the study subjects in the training and testing data for each fracture type is listed in Table 1.

**Table 1:** Fractures and the corresponding number of patients in the training and testing data

Fractures	# Patients in Training	# Patients in Testing	Total
Ankle	90	76	166
Clavicle	32	26	58
Distal Forearm	102	86	188
Face	60	52	112
Feet and Toes	185	158	343
Hand and Fingers	140	120	260
Other Spine	28	24	52
Patella	10	8	18
Pelvis	62	52	114
Proximal Femur	74	62	136
Proximal Humerus	48	40	88
Ribs	104	90	194
Scapula	9	8	17
Shaft and Distal Femur	13	10	23
Shaft and Distal Humerus	13	12	25
Shaft and Proximal Radius/Ulna	41	36	77
Skull	4	4	8
Sternum	5	4	9
Tibia and Fibula	37	32	69
Vertebral Body	215	184	399

## The rule-based NLP algorithm

**Fig. 1:**
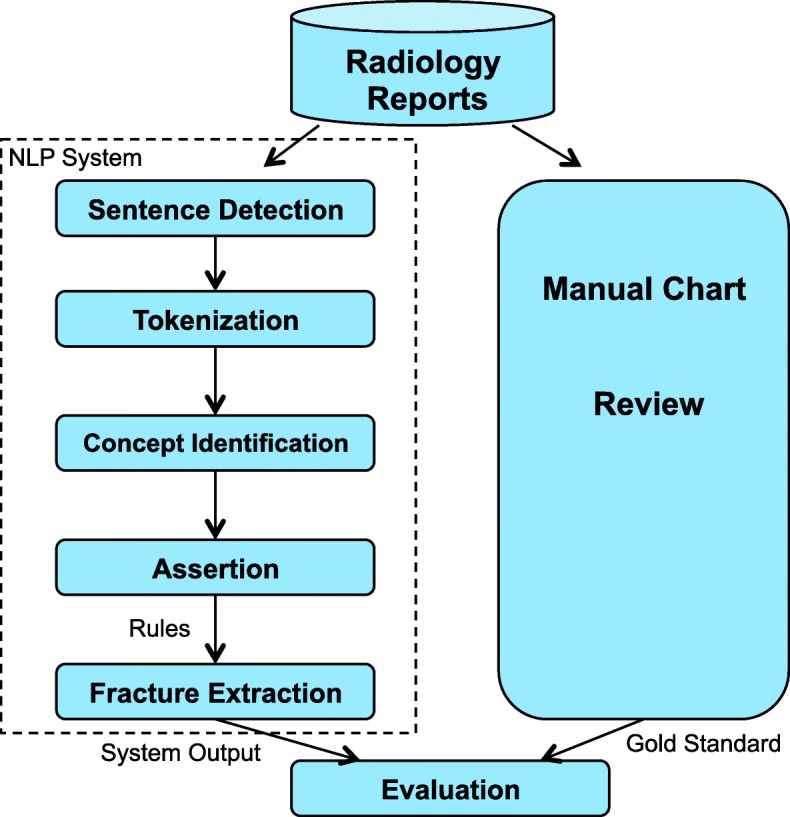
Study Design

Figure 1 shows the overall design of the study. The rule-based NLP algorithm was developed using Medtagger, an NLP tool developed based on the Apache Unstructured Information Management Architecture (UIMA) pipeline [23], to facilitate information extraction from clinical narratives. Based on the training data, we developed rules for identifying each fracture type according to physicians’ knowledge and experience, and refined these rules via verification with physicians. These rules were also supplemented with historical rules developed by the Osteoporosis Research Program to aid the nurse abstractors in fracture identification.

The regular expressions in our NLP algorithm for each fracture are listed in Table 2 and the fracture modifiers are listed in Table 3. MedTagger uses the rules within detected sentences to identify a specific fracture type. The rules are “\b(%reFractureModifier).*(%reFractureCategory)\b” or “\b(%reFractureCategory). *(%reFractureModifier)\b” where reFractureCategory represents regular expressions for the specific fracture category in Table 2 and reFractureModifier modifiers in Table 3. During the interactive refinement of NLP algorithm with physicians, we also added a few exclusion rules to reduce the number of false positives in the training data. For example, if keywords, such as “rule out” or “r/o”, and “negative” occurred in the sentence, we excluded the extracted fractures. Finally the rule-based NLP algorithm was evaluated on the held-out testing data.

**Table 2:** Regular Expressions in the rule-based NLP algorithm for the identification of fractures

Fractures	Regular Expressions
Ankle	(inversion)?ankle |tillaux|bimalleolar |distal.*(fibular|tibial) |dupuytren’s |(lateral|medial|posterior) malleolus |Pott’s |trimalleolar
Clavicle	(shaft|acromial end) of clavicle |interligamentous |collar bone |clavic(le|al) |clav |
Distal Forearm	barton’s |colles’ |(distal|metaphyseal).*(wrist|radius|radial|ulna|ulnar|forearm) |smith’s |styloid process |head of ulna(r)? |ulna(r)? head |wrist
Face	(inferior)?maxilla(ry)? |nasal |(upper|lower)?jaw |orbit(al)? |malar bone |palate |mandible |zygoma(tic)? |mandibular(ramus)? |facial |naso-orbital
Feet and Toes	(meta)?tarsal |astragalus |instep |calcaneus |os calcis |navicular |cuboid |cuneiform |talonavicular ossicle |heel |talus |phalan(x|ges?) |toe
Hand and Fingers	hand |fingers? |(meta)?carpals? |mc |(hand |finger) phalanges |(proximal|distal|middle).*phalanx |capitate |hamate |lunate |scaphoid |navicular |trapezi(um|id) |pisiform |triquetrum |metacarpus |bennett’s |thumb |sesamoid |boxer’s |bar room
Other Spine Fractures	vertebra(e|l) |cervical vertebrae |posterior elements of vertebrae |coccyx |spinous process |neural arch |transverse process |spine |pedicle |C(1|2|3|4|5|6)
Patella	knee ?(cap|pan) |patella(r)?
Pelvis	acetabulum |acetabular |pelvic rim |ilium |pubis |pubic |innominate |rami |ischium |ischial |sacrum |sacral |obturator ring |pelvi(c|s)
Proximal Femur	(femoral |femur)(head|neck) |(trans)?cervical |(sub)?capital |intracapsular |trans(|-)?epiphyseal |base of neck |basilar femoral neck |cervicotrochanteric |(greater|lesser) trochanter |(inter|per|intra)trochanteric
Proximal Humerus	(anatomical|surgical)? (head|neck|head(-|/)neck|neck(-|/)head) |(humerus|humeral) (|shoulder |proximal end) |extra ?capsular |(humerus|humeral).*(head|neck|head(-|/)neck|neck(-|/)head) |head of (humerus|humeral) |(greater|lesser)? tuberosity |proximal humerus |humerus proximal
Ribs	rib(s)? |(rib|thoracic) cage
Scapula	acromion|coracoid(process)? |scapula |glenoid(cavity|fossa)? |shoulder blade
Shaft and Distal Femur	diaphyseal fracture of femur |subtrochanteric |(lateral|medial) condylar |supracondylar |(shaft|lower end) of femur |mid femur
Shaft and Distal Humerus	elbow |condylar |shaft of (humerus|humeral) |(distal|end of|shaft).*(humerus|humeral) |supracondylar |epicondyle
Shaft and Proximal Radius/Ulna	proximal.*(forearm|radius|radial|ulna(r)?) |coronoid process |metaphyseal of (the)?proximal.*(forearm|radius|radial|ulna(r)?) |(radius|ulna) diaphyseal |Monteggia(’s)? |Dupuytren(’s)? |(neck|head|head(-|/)neck|neck(-|/)head) of.*(radius|radial) |(radius|radial) (neck|head|head(-|/)neck|neck(-|/)head) |Galeazzi(’s)? |shaft (of)? (ulna(r)? |radius)|radial shalf |ulna(r)? shaft |metadiaphyseal |olecranon(process)?
Skull	(base|vault) of the skull |vault |(ethmoid|sphenoid) (sinus|base) |sphenoid |occipital |vertex skull |calvaria(l)? |calvarium
Sternum	breast()?bone |sternum |manubrium |xyphoid
Tibia and Fibula	(proximal)?fibula |intercondylar eminence shaft |(lateral|tibia |fibula) condyle |lateral tibial plateau |((medial)?tibia |tibial) shaft |tuberosity
Vertebral Body	ballooning (of inter ?spaces?)? |biconcave |burst |axis |cod-fish |endplate |loss of height ||(t|l)-?spine |lumbar |thoracic |collapse |l(1|2|3|4|5) |t(1|2|3|4|5|6|7|8|9|10|11)

**Table 3:** Fracture modifiers

(micro-?)?fracture(s|d)? |separation |fxs? |broken |cracked |displace(d)? |fragment	

## Evaluation

We calculated the overall agreement between the proposed NLP algorithm and the gold standard. Five metrics, namely sensitivity, specificity, positive predictive value (PPV), negative predictive value (NPV) and F1-score, were used to measure the performance of the NLP system for each fracture, and micro-averaged values of these metrics were used to evaluate the overall performance. The definitions of these metrics are as follows:

$$\begin{aligned} Sensitivity&=\frac{TP}{TP+FN}, Specificity=\frac{TN}{TN+FP},\\ PPV &= \frac{TP}{TP+FP}, NPV=\frac{TN}{TN+FN}, \\F1\text{-}score&=\frac{2 PPV\cdot Sensitivity}{PPV+Sensitivity},\\ {Sensitivity}_{micro}&=\frac{{\sum\nolimits}_{i} {TP}_{i}}{{\sum\nolimits}_{i} {TP}_{i}+{FN}_{i}},\\ {Specificity}_{micro}&=\frac{{\sum\nolimits}_{i} {TN}_{i}}{{\sum\nolimits}_{i} {TN}_{i}+{FP}_{i}},\\ {PPV}_{micro}&= \frac{{\sum\nolimits}_{i} {TP}_{i}}{{\sum\nolimits}_{i} {TP}_{i}+{FP}_{i}},\\ {NPV}_{micro}&=\frac{{\sum\nolimits}_{i} {TN}_{i}}{{\sum\nolimits}_{i} {TN}_{i}+{FN}_{i}},\\ F1\text{-}{score}_{micro}&=\frac{2 {PPV}_{micro}\cdot {Sensitivity}_{micro}}{{PPV}_{micro}+{Sensitivity}_{micro}}, \end{aligned} $$

where TP, TN, FP, and FN represent true positives, true negatives, false positives, and false negatives, respectively, and *i*=1,2,…,20 is the *i*th fracture type.

## Results

Table 4 shows the experimental results of the NLP algorithm. Overall the NLP algorithm has a high micro-average F1-score of 0.961, which validates the effectiveness of the proposed NLP algorithm for identifying the twenty skeletal site-specific fractures from the radiology reports. The micro-average PPV and specificity are 1.0 and 1.0, respectively, which shows that the NLP algorithm has high precision in identifying positives and negatives. The micro-average sensitivity is 0.930, which implies that the rules in the NLP algorithm are sufficient in identifying fractures. 8 fracture types (40%) have obtained F1-scores of 1.0 while a total of 17 fracture types (85%) F1-scores of above 0.9 (including 1.0). The lowest F1-score is to extract vertebral body fractures (F1-score =0.806).

**Table 4:** Experimental results of the NLP algorithm for each fracture type

Fractures	Sensitivity	Specificity	PPV	NPV	F1-score
Ankle	0.974	1.000	1.000	0.974	0.987
Clavicle	1.000	1.000	1.000	1.000	1.000
Distal Forearm	1.000	1.000	1.000	1.000	1.000
Face	0.760	1.000	1.000	0.806	0.864
Feet and Toes	0.960	1.000	1.000	0.962	0.980
Hand and Fingers	0.918	1.000	1.000	0.924	0.957
Other Spine Fractures	0.875	1.000	1.000	0.889	0.933
Patella	1.000	1.000	1.000	1.000	1.000
Pelvis	0.952	1.000	1.000	0.955	0.976
Proximal Femur	1.000	1.000	1.000	1.000	1.000
Proximal Humerus	1.000	1.000	1.000	1.000	1.000
Ribs	0.933	1.000	1.000	0.938	0.966
Scapula	1.000	1.000	1.000	1.000	1.000
Shaft and Distal Femur	0.800	1.000	1.000	0.833	0.889
Shaft and Distal Humerus	0.857	1.000	1.000	0.875	0.923
Shaft and Proximal Radius/Ulna	0.952	1.000	1.000	0.955	0.976
Skull	1.000	1.000	1.000	1.000	1.000
Sternum	1.000	1.000	1.000	1.000	1.000
Tibia and Fibula	0.944	1.000	1.000	0.947	0.971
Vertebral Body	0.675	1.000	1.000	0.755	0.806
Micro-Average	0.930	1.000	1.000	0.941	0.961

Here we provide a few examples of false positives and false negatives during training, and analyze why the NLP algorithm failed in these cases. The NLP algorithm was unable to identify ankle fracture for **Patient A** since the indication term “debride” that rarely appeared in the training data was not considered in the rules. The same situation happened for **Patient B** who had face fracture but the NLP algorithm failed to identify due to the missing keyword “lamina papyracea” in the rules. Some false positives and false negatives fundamental problems in NLP, such as sentence boundary detection and negation detection. For example, the algorithm failed to detect the sentence starting from “superior” in **Patient C**’s clinical note. The algorithm failed to detect the negation for **Patient D**. Thus, we added rules for boundary detection and terms for negation that were specific to our clinical note corpus.

**Patient A**: *Exam: Fluoro Assistance less < 1hr Indications: left ankle debride ORIGINAL REPORT ? DATE Mobile image intensifier used. Electronically signed by: PHYNAME. DATE.*

**Patient B**: *CT examination of the head and maxillofacial bones performed without IV contrast demonstrates a mildly displaced fracture of the superior right lamina papyracea.*

**Patient C**: *No inflammatory changes to suggest cholecystitis superior endplate compression fractures of T11 and T12 vertebral body*

**Patient D**: *The bone scan was negative for an acute fracture at that area, although an acute fracture in the vertebral body of L1 was noted.*

Some terms are clinically ambiguous. For example, the term “phalanx” is ambiguous since it could refer to either a finger or a toe. Based on the training data, we added modifiers “proximal/distal/middle” to “phalanx” for hand and fingers fractures. A better solution might be using the metadata of radiology notes to pre-identify whether the X-ray is for hand or foot.

Some false negatives are due to the co-reference in the report. For example, **Patient E** was not identified due to that the term “findings” is co-referenced to the hand fractures. Some false negatives are due to the ambiguity or incorrect negation detection. For example, **Patient F** had vertebral body fracture based on the meaning of sentence but was incorrectly classified as negated.

**Patient E**: *Cortical irregularity of the dorsal aspect of the distal tuft of the left thumb. Findings likely represent a small fracture.*

**Patient F**: *It does not appear the L1 compression fracture is the cause of her pain.*

## Discussion

We have developed a rule-based NLP algorithm for the identification of twenty skeletal site-specific fractures from radiology reports. We have validated its effectiveness using the radiology reports of a community-based cohort at Mayo Clinic. The NLP algorithm could be utilized to accurately identify the patients with fractures and those who are also at high risk of future fractures due to osteoporosis. Appropriate care interventions to those patients, not only the most at-risk patients but also those with emerging risk, would significantly reduce future fracture. This would particularly help transition the current form of fee-for-service care to value-based care since it might be difficult to make impactful interventions for the real high-risk category of patients while more significant to focus on the emerging-risk category in an attempt to keep them from becoming high risk [24].

Recently, machine learning techniques have shown promise for automated outcome classification, particularly when large volumes of data are available [8]. Since the rules in the NLP algorithm need to be laboriously fine-designed through interactive verifications between rule designers and physicians, machine learning provides a solution that significantly reduces or eliminates the workload of designing rules. One of our ongoing works is to apply machine learning classifiers and advanced deep learning methods to tackle the fracture classification task [8, 25]. However, the rule-based NLP algorithm is straightforward to interpret for physicians and easy to be modified through interactive refinement with physicians’ feedbacks. As shown by [6], only one-third of the vendors relied entirely on machine learning, and the systems developed by large vendors, such as IBM, SAP, and Microsoft, are completely rule-based. An additional benefit we observed was that the NLP algorithm augmented the guideline for manually annotating fractures as many keywords from the algorithm had been added in the guideline. For example, “clav fx” has been added to the guideline of abstracting clavicle fracture; “inferior maxillary”, “zygomatic”, “facial” and “naso-orbital” have been added for face fracture; “C1”-“C6” have been added for other spine fractures; and “acetabular”, “sacral”, “ischial”, “pubic” have been added for pelvis fracture.

This study has two limitations. First, we only verified the effectiveness of NLP algorithm on radiology reports. It would be interesting to evaluate the NLP algorithm on other free-text EHR resources, such as clinical notes. Second, we only tested the NLP algorithm in one institution. It is also interesting to study the portability of the NLP algorithm across institutions with disparate sublanguages [26].

## Conclusions

In this study, we developed a rule-based NLP algorithm for identification of twenty skeletal site-specific fractures from radiology reports. The keywords and regular expressions in the comprehensive NLP algorithm could be reused in different fracture identification applications. Our empirical experiments validated the effectiveness of the NLP algorithm using the radiology reports of a community-based cohort at Mayo Clinic. The micro-averaged results of the NLP algorithm for the twenty fractures are 0.930, 1.0, 1.0, 0.941, 0.961 in terms of sensitivity, specificity, PPV, NPV, and F1-score, respectively. 8 fracture types (40%) have obtained F1-scores of 1.0 while a total of 17 fracture types (85%) F1-scores of above 0.9. The results verified the effectiveness of the proposed rule-based NLP algorithm in automatic identification of fractures from radiology reports.

## Abbreviations

CT: Computed tomography
EHRs: Electronic health records
IRB: Institutional review board
NLP: Natural language processing
NPV: Negative predictive value
PPV: positive predictive value
REP: Rochester epidemiology project
UIMA: Unstructured information management architecture
WHO: World health organization
